# Antigenic evolution of H3N2 influenza A viruses in swine in the United States from 2012 to 2016

**DOI:** 10.1111/irv.12610

**Published:** 2018-10-07

**Authors:** Marcus J. Bolton, Eugenio J. Abente, Divya Venkatesh, Jered A. Stratton, Michael Zeller, Tavis K. Anderson, Nicola S. Lewis, Amy L. Vincent

**Affiliations:** ^1^ Virus and Prion Research Unit National Animal Disease Center USDA‐ARS Ames Iowa; ^2^ Department of Zoology University of Cambridge Cambridge UK; ^3^ Department of Veterinary Diagnostic and Production Animal Medicine College of Veterinary Medicine Iowa State University Ames Iowa; ^4^ Present address: Department of Pathobiology and Population Sciences Royal Veterinary College Hatfield UK

**Keywords:** antigenic cartography, antigenic evolution, H3N2, influenza A virus, swine

## Abstract

**Background:**

Six amino acid positions (145, 155, 156, 158, 159, and 189, referred to as the antigenic motif; H3 numbering) in the globular head region of hemagglutinin (HA1 domain) play an important role in defining the antigenic phenotype of swine Clade IV (C‐IV) H3N2 IAV, containing an H3 from a late 1990s human‐to‐swine introduction. We hypothesized that antigenicity of a swine C‐IV H3 virus could be inferred based upon the antigenic motif if it matched a previously characterized antigen with the same motif. An increasing number of C‐IV H3 genes encoding antigenic motifs that had not been previously characterized were observed in the U.S. pig population between 2012 and 2016.

**Objectives:**

A broad panel of contemporary H3 viruses with uncharacterized antigenic motifs was selected across multiple clades within C‐IV to assess the impact of HA1 genetic diversity on the antigenic phenotype.

**Methods:**

Hemagglutination inhibition (HI) assays were performed with isolates selected based on antigenic motif, tested against a panel of swine antisera, and visualized by antigenic cartography.

**Results:**

A previously uncharacterized motif with low but sustained circulation in the swine population demonstrated a distinct phenotype from those previously characterized. Antigenic variation increased for viruses with similar antigenic motifs, likely due to amino acid substitutions outside the motif.

**Conclusions:**

Although antigenic motifs were largely associated with antigenic distances, substantial diversity among co‐circulating viruses poses a significant challenge for effective vaccine development. Continued surveillance and antigenic characterization of circulating strains is critical for improving vaccine efforts to control C‐IV H3 IAV in U.S. swine.

## 1 INTRODUCTION

Influenza A virus (IAV) is an important respiratory pathogen of both humans and swine. Vaccination is the main strategy employed to control the morbidity and mortality associated with IAV illnesses in both hosts. Although vaccine platforms, formulations, and strain selection processes differ between host species, all widely utilized IAV vaccines primarily target the hemagglutinin (HA) protein. While IAV vaccine strain selection for seasonal human vaccines is a globally coordinated event led by the World Health Organization with strains publicly announced twice per year,^1^ veterinary vaccine companies are not required to report virus strain names on vaccine labels or product inserts included in commercially available vaccines. Fully licensed vaccines for commercial use in the United States include two killed multivalent vaccines, an alphavirus‐vectored vaccine, and a newly licensed live‐attenuated influenza A virus vaccine (LAIV).^2^, ^3^, ^4^ Furthermore, swine producers may use farm or company‐specific custom autogenous vaccines to protect their swine herds from IAV in swine (IAV‐S).^5^, ^6^ These factors limit the ability to evaluate vaccine strain matching and efficacy from a national perspective.

H3N2 was recognized in the swine population in 1999 with an HA from Fujian‐like human seasonal IAV.^7^, ^8^ These H3N2 evolved into what was later termed Clade IV (C‐IV) based on phylogenetic analysis, and C‐IV IAV‐S has continued to circulate in North America since approximately 2005. In addition to antigenic drift observed during the sustained circulation of this introduction, other spillover events of human seasonal H3 IAV occur periodically, further increasing the antigenic diversity of H3 viruses in the U.S. swine population. A more recent human seasonal H3 HA was detected in pigs as a novel interspecies transmission event, and there is evidence of persistence in the swine population.^9^ It is unclear if the antigenic evolution of these recent human‐like HA's will follow similar patterns as the C‐IV H3 IAV‐S or if this genetically and antigenically distinct human‐like H3 HA will displace the endemic C‐IV H3 HA in the United States, but at present time, the two H3 IAV‐S clades co‐circulate. Previous studies in search of molecular determinants responsible for antigenic drift in human H3 IAV identified seven amino acid positions (145, 155, 156, 158, 159, 189, and 193; H3 numbering throughout) in the HA protein that largely determined the antigenic phenotype.^10^, ^11^ A similar study with H3 IAV‐S found that six of the seven positions (145, 155, 156, 158, 159, and 189) implicated in human IAV antigenic evolution were also important for the antigenic phenotype of C‐IV H3 IAV‐S, and phenotypic differences were observed among co‐circulating swine IAV.^12^ Analysis by antigenic cartography revealed distinct antigenic groupings of viruses, termed antigenic clusters, and labeled by different colors for visualization. The antigenic clusters were associated with specific combinations of amino acids at the six positions, and, thus, the combinations of these key positions were referred to as an “antigenic motif.” For example, the Cyan antigenic cluster was comprised of viruses encoding at least four antigenic motifs (NHNNYR/NHNDYR/NNNDYR/NHSYR), and the Red antigenic cluster was comprised of at least four antigenic motifs (NYNNYK/NYHNYK/NYNNHK/NHNNYK). Site‐directed mutagenesis at these six amino acid positions in a prototype C‐IV H3 IAV‐S strain confirmed that these six positions played a key role in defining the antigenic phenotype.^13^ Trends in antigenic motif patterns over time revealed a predominance of viruses encoding Cyan antigenic cluster motifs in 2009, followed by a steady decline. The emergence of viruses encoding Red antigenic cluster motifs was observed in 2010, followed by sustained circulation through 2016, and the emergence of viruses encoding Green antigenic motifs in 2013 (previously “light green”^13^). However, the previous study reported that 23% of virus isolates collected from 2009 to 2015 encoded antigenic motifs that had yet to be antigenically characterized. These uncharacterized H3 IAV‐S likely represented additional antigenic diversity.

In this study, we selected contemporary C‐IV H3 IAV‐S isolates in the US based on the observed expanding genetic diversity of the HA gene and an increase in antigenic motif patterns. The antigenic phenotype of 50 C‐IV H3 IAV‐S collected between 2012 and 2016 was characterized using hemagglutination inhibition (HI) assay data generated with swine antisera and visualized with antigenic cartography. The selected viruses contained uncharacterized motifs to determine the impact of amino acid substitutions at the six key sites as well as viruses with previously characterized antigenic motifs to validate previous observations.

## 2 METHODS

### 2.1 Sequence analyses

1358 swine H3 HA protein sequences isolated between 2012 and 2016 in the United States were downloaded from the Influenza Research Database (IRD)^14^ on July 3, 2017. Viruses that were not C‐IV or were identified as a duplicate sequence isolated on the same day in the same U.S. state were removed (n = 351). The resulting 1007 HA sequences were aligned with MUSCLE using default settings within Geneious (v10.3.2),^15^, ^16^ and amino acids at positions 145, 155, 156, 158, 159, and 189, defined as the antigenic motif, were recorded to determine the frequency of antigenic motifs over time.

A maximum‐likelihood phylogeny was inferred from the protein alignment using FastTree (v2.1) with default settings with a JTT+CAT model of molecular evolution.^17^ Each HA protein sequence was assigned to one of six clades within C‐IV (clades A‐F) following Kitikoon et al,^18^ or to the recently emerged human‐like clade.^9^

C‐IV H3N2 IAV‐S (n = 1007) were grouped by antigenic motif and at least three strains were selected for analysis from uncharacterized antigenic motif groups. For each selected motif, a strain was chosen for both high and low similarity to the motif group consensus HA sequence. A third strain was selected to assess common substitution patterns within the given antigenic motif group if present. Additional strains were selected with HAs that encoded less frequently detected antigenic motifs. A total of 50 C‐IV H3N2 IAV‐S were selected as antigens for hemagglutination inhibition (HI) assays and/or antiserum production (Table S1).

### 2.2 Viruses

Selected viruses were obtained from the U.S. Department of Agriculture (USDA) voluntary IAV‐S surveillance system repository held at the National Veterinary Services Laboratories in Ames, IA. Viruses were propagated in Madin‐Darby canine kidney (MDCK) cells grown in Opti‐MEM (Thermo Fisher Scientific, Waltham, MA, USA) supplemented with antibiotic‐antimycotic (Thermo Fisher Scientific).

### 2.3 Antiserum production

Swine antiserum was produced by immunizing two pigs as previously described.^12^ For use in HI assays, sera were incubated at 37°C overnight with receptor‐destroying enzyme (RDE(II); Denka Seiken, Tokyo, Japan). After the addition of 0.85% saline (w/v) the following morning, sera were incubated at 56°C for 45 min to deactivate the RDE, followed by adsorption with 50% turkey red blood cells at 4°C to remove any additional nonspecific inhibitors of HA.

### 2.4 Hemagglutination inhibition assays

Standard HI assays were performed with turkey red blood cells, and fold reduction values for endpoint titers were calculated by dividing the homologous geometric mean titer (GMT) for each pair of sera by the heterologous GMT of each test antigen.

### 2.5 Antigenic cartography

HI data generated in this study were merged with a subset of H3 IAV‐S HI data generated previously by Lewis et al using the same methods described herein (Table S2).^12^ Antigenic relationships were visualized in multi‐dimensional space using antigenic cartography.^12^, ^19^, ^20^

## 3 RESULTS

### 3.1 Contemporary C‐IV IAV‐S strains encoded antigenic motifs not previously characterized

**Figure 1:**
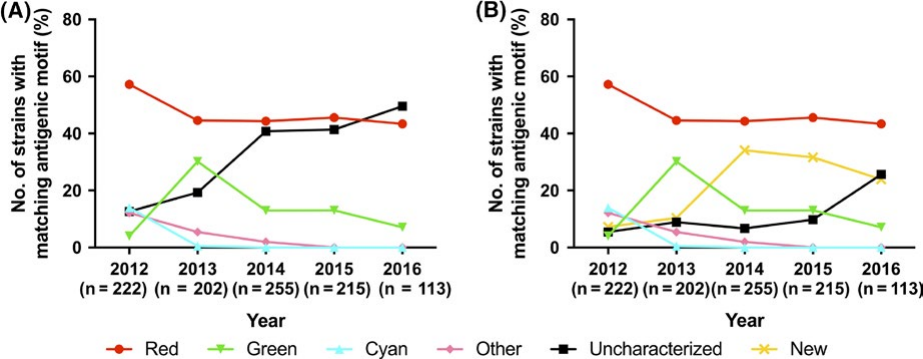
Temporal frequency of H3 antigenic clusters. (A) Temporal frequency of H3 antigenic clusters prior to this study. (B) Temporal frequency of H3 antigenic clusters following this study. Cluster designations and coloring follow the color scheme used previously by Lewis et al.^12^ Strains denoted “Other” encode outlier antigenic motifs of low prevalence. Strains encoding an antigenic motif not yet phenotypically characterized are denoted as “Uncharacterized”, while “New” strains encode an antigenic motif characterized in this study

Although there were 73 unique antigenic motifs detected, 90% of the HA sequences encoded one of the 20 most frequently detected motifs (904/1007) (Table 1). The two most frequently detected motifs in H3 IAV‐S over the five‐year timespan were NYNNYK and KYNNYK, corresponding to the previously defined Red and Green antigenic clusters, respectively.^12^ However, 31.4% of the HA genes collected between 2012 and 2016 encoded a previously uncharacterized antigenic motif,^12^, ^13^ including six of the top 10 motifs (Figure 1A). More recently, 43% of viruses from 2014 to 2016 encoded an uncharacterized antigenic motif, roughly equivalent to the frequency of the predominant Red antigenic cluster (45%). This high percentage of viruses encoding an uncharacterized motif revealed a lack in knowledge of the antigenic diversity of H3 IAV‐S in the US.

**Table 1:** Twenty most frequently encoded antigenic motifs from Clade IV virus strains collected 2012‐2016 (n = 904)

Antigenic motif	No. of virus strains with motif	Putative antigenic cluster	No. of isolates tested^a^^,^ b
NYNNYK	445	Red	8
KYNNYK	139	Green	12
NYSNYK	77	Unknown	7
KYHNNK	33	Unknown	4
NHNNYR	30	Cyan^c^	
SYKNYK	25	Unknown	4
KHNNHK	25	Unknown	5
NHNNYK	19	Red	1
KYNNSK	18	Unknown	2
KYHNYK	13	Unknown	2
KHKNYS	10	Purple	
KHHNNK	10	Unknown	2
NYKNYS	9	Unknown	
NHNNHK	9	Unknown	1
KYNNNK	8	Gold	
NYHNYK	8	Red	
KHNNYK	7	Blue	
NYHGHE	7	Brown	
KYHDYK	6	Unknown	
NYKNYK	6	Unknown	1

a Number of isolates tested in this study.

b Two additional isolates were tested that did not encode a motif listed here.

c Previously characterized and not detected in 2014‐2016.

### 3.2 Uncharacterized antigenic motifs represented additional antigenic diversity

**Figure 2:**
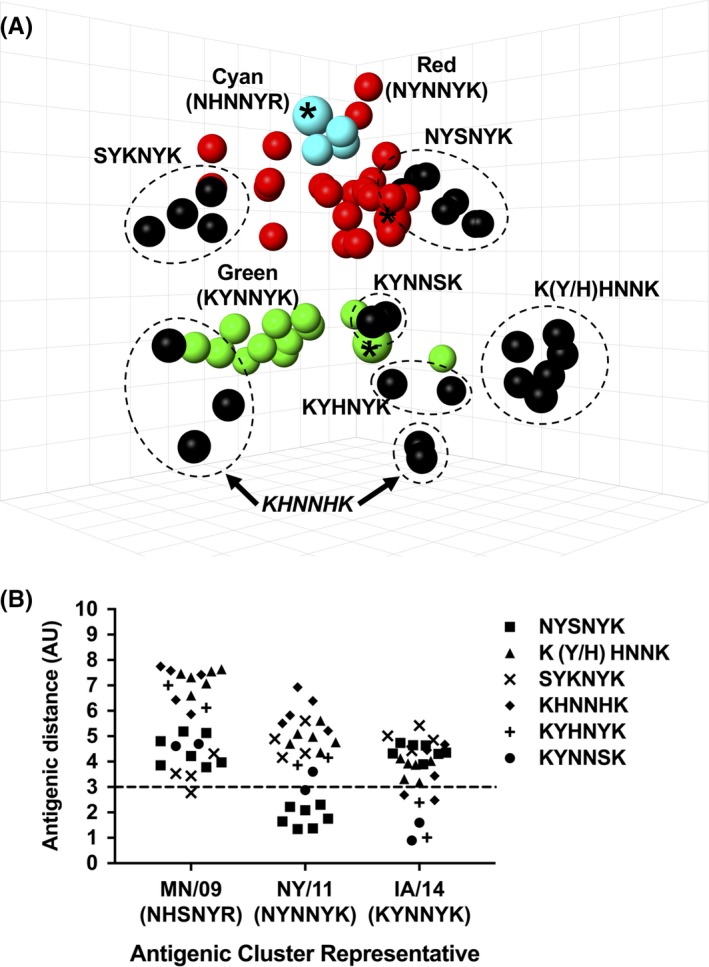
Antigenic phenotype of strains encoding a previously uncharacterized motif. (A) Three‐dimensional antigenic map of strains encoding a previously uncharacterized motif. Viruses encoding identical antigenic motifs are grouped (dotted circles) and labeled. Predominant antigenic clusters from 2009 to 2016, with cluster representative viruses denoted by an asterisk (*), are visualized for reference (the dominant antigenic motif is indicated for each colored phenotype). (B) Antigenic distance from Cyan (MN/09), Red (NY/11), and Green (IA/14) cluster representative strains. The 3 antigenic unit (AU) line denotes an 8‐fold loss in HI cross‐reactivity, the cutoff typically used in human H3 IAV antigenic studies to define significant antigenic drift

Twenty‐nine viruses encoding ten previously uncharacterized antigenic motifs from three distinct phylogenetic clades of C‐IV H3 IAV‐S were selected for characterization (Table S1). The HI results were used to generate an antigenic map (Figure 2A), and strains encoding the same archetypal motif generally clustered together in the antigenic map with one exception described below. A/swine/Minnesota/02782/2009 (MN/09, Cyan), A/swine/New York/A01104005/2011 (NY/11, Red), and A/swine/Iowa/A01480656/2014 (IA/14, Green) were chosen as reference viruses for the three major antigenic clusters on the criteria of (a) similarity to a cluster's HA consensus sequence, (b) close proximity to cluster centroid in the antigenic map, and (c) tested previously for comparison with the newly generated data. Antigenic distance of viruses with emerging motifs was then compared on a pairwise basis to the reference strains.

All viruses encoding an NYSNYK motif were <3 AU from NY/11 (NYNNYK), the Red cluster representative, indicating contemporary strains encoding the NYSNYK motif grouped within the Red antigenic cluster. Despite the intra‐cluster variation among antigenic motifs, N145, Y159, and K189 were conserved among the Red antigenic cluster viruses (Figure 2B). As previously observed in the Red cluster, variation at positions 155 and 156 alone was not phenotypically significant. Therefore, the Red cluster motif in the C‐IV genetic background was composed of four antigenic motifs (NYNNYK, NHNNYK, NYHNYK, and NYSNYK).

Antigens encoding either a KYNNSK or KYHNYK motifs were mapped nearest to the Green antigenic cluster (previously associated with only a KYNNYK antigenic motif), with antigenic distances ranging 0.9‐2.4 AU from IA/14. Therefore, the Green cluster phenotype included at least three antigenic motifs (KYNNSK, KYHNYK, or KYNNYK) with conserved residues at K145, Y155, N158, and K189 currently defining this antigenic phenotype.

Antigens encoding KYHNNK or KHHNNK antigenic motifs, color‐coded as Peach in this study, formed a new antigenic cluster distinct from previously described clusters. Peach antigens were positioned more than 3 AU (range of 3 to 4 AU) from the Green representative IA/14 (KYNNYK) (Figure 2B). The novel Peach antigenic motifs differed at position 159 when compared to the Green antigenic motifs, indicating an important role in the context of the Peach antigenic motif.

Antigens encoding an SYKNYK motif also formed a putative antigenic cluster, as these antigens ranged from 2.8 to 4.3 AU away from the nearest Cyan cluster representative MN/09 (NHSNYR). However, SYKNYK antigens also overlapped with the range of antigenic space occupied by Red antigens and were not detected in 2016 or 2017, so were not given a unique cluster designation at this time. SYKNYK antigens differed from the Cyan and Red cluster antigens by combinations of substitutions at positions 145, 155, 156, and 189. A single antigen was tested for each of the following motifs: NHNNHK, mapping nearest to Cyan (3.2 AU from MN/09); NYKNYK, mapping nearest to Cyan (4.1 AU from MN/09); and NYKNYN, nearly equidistant from each cluster representative (NY/11, 3.3 AU; MN/09, 3.5 AU; IA/14, 3.3 AU) (data not shown).

Viruses with HA containing a KHNNHK motif were the exception to the trend of similar antigenic motifs clustering together in the antigenic map. Antigens encoding KHNNHK motifs did not form a cohesive cluster but mapped nearest to the Green antigenic cluster, with antigenic distances ranging from 2.5 to 4.7 AU from the Green representative IA/14. Viruses encoding KHNNHK were likely distinct from Green cluster viruses as a result of substitutions at positions 155 and 159. Additional amino acid differences outside the antigenic motif in these HA included positions 131, 150, 192, 196, and 223, which may play a role in the antigenic differences observed.

Following the expanded antigenic motif designations of the Red and Green clusters, and the new designation of Peach, we re‐calculated the frequencies of putative antigenic clusters, demonstrating a marked decrease in the percent of uncharacterized motifs in the years 2014 and 2015 (7%‐10%) (Figure 1B). However, the number of uncharacterized motifs increased again in 2016, suggesting continued potential for antigenic variation.

### 3.3 Antigenic motif alone was not sufficient to explain intra‐cluster diversity

**Figure 3:**
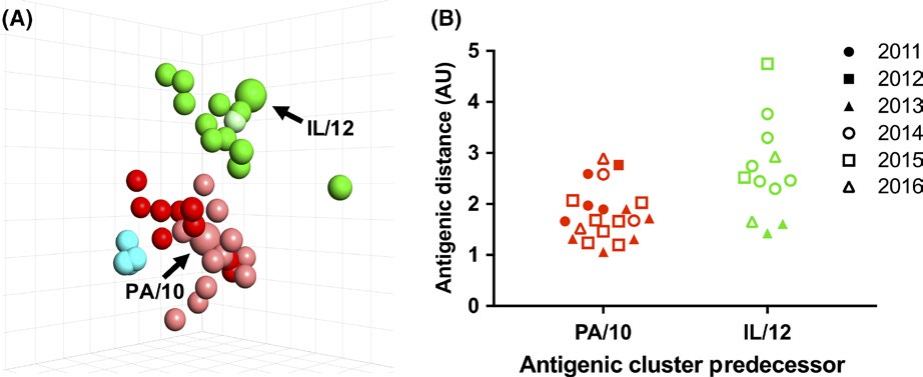
Antigenic evolution within antigenic clusters. (A) Three‐dimensional antigenic map of strains encoding a Red or Green antigenic motif. Newly characterized viruses encoding KYNNYK (bright green) or NYNNYK (bright red), along with a previously characterized Green virus (pale green) and previously characterized Red (pale red) and Cyan (cyan) cluster viruses. (B) Intra‐cluster antigenic distance from Red (PA/10) and Green (IL/12) cluster predecessors across the study time frame. One antigenic unit (AU) is equal to a twofold loss in cross‐reactivity

To determine whether variation outside of the 6 amino acid motif positions contributed to intra‐cluster drift, we selected strains encoding the most prevalent antigenic motifs corresponding to the Red and Green antigenic clusters with collection dates from 2012 to 2016. Eight viruses encoding an NYNNYK antigenic motif and one virus encoding an NHNNYK motif were chosen from the Red antigenic motifs (Table S1, Figure 3). Twelve viruses encoding a KYNNYK antigenic motif were similarly tested among viruses encoding Green antigenic motifs (Table S1, Figure 3). All newly characterized viruses mapped relatively near previously tested viruses encoding the same motif, but with demonstrable intra‐cluster variation (up to 4.8 AU for Red and up to 6.3 AU for Green) (Figure 3A). To study temporal intra‐cluster drift, A/swine/Pennsylvania/A01076777/2010 (PA/10) and A/swine/Illinois/A01327903/2012 (IL/12) were chosen as reference antigens because they were the earliest antigens characterized within the Red and Green antigenic clusters, respectively. The antigenic distances of each virus to its respective cluster predecessor were plotted to observe intra‐cluster diversity over time (Figure 3B). Virus strains within the Green antigenic cluster demonstrated greater intra‐cluster variation and antigenic distance from the cluster representative strain over a four‐year period than virus strains belonging to the Red antigenic cluster over a six‐year period.

### 3.4 Antisera from predominant antigenic clusters did not effectively cross‐react with viruses containing divergent motifs

**Figure 4:**
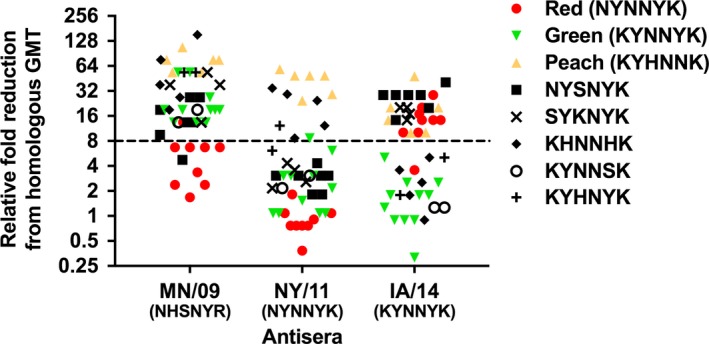
Cross‐reactivity of swine sera raised against antigenic cluster representatives. Relative fold reduction in heterologous strains from antisera raised to MN/09 (Cyan), NY/11 (Red), and IA/14 (Green). A ≥ 8‐fold reduction in the heterologous GMT from the homologous reaction is considered a significant loss in cross‐reactivity by the sera. The dominant antigenic motif is indicated for colored phenotypes

Monovalent vaccine antisera raised against viruses of three predominant antigenic clusters (Cyan, Red, and Green) were tested in HI assays against currently circulating strains and pairwise fold reduction in each heterologous geometric mean titer (GMT) to the homologous GMT for the representative strains were compared. A ≥ 8‐fold reduction defined significant loss in cross‐reactivity. Sera against the selected Red and Green representatives remained cross‐reactive with strains encoding their own motifs, but the Red NY/11 antisera demonstrated broader cross‐reactivity to heterologous Green strains than the converse (Figure 4). Viruses encoding the Red antigenic motif demonstrated between a 0.4‐1.8‐fold reduction in GMT compared to the homologous GMT of NY/11 (Figure 4). A 0.9‐5.0‐fold reduction in GMT with IA/14 antisera was observed for viruses encoding the Green antigenic motif (Figure 4). Despite moderate intra‐cluster antigenic variation within the Red and Green antigenic clusters between 2012 and 2016, within‐cluster cross‐protection might be retained.

Sera raised against cluster representatives were less cross‐reactive with strains encoding heterologous motifs from other antigenic clusters. Sera raised against the Cyan cluster representative MN/09 cross‐reacted with 8/9 of the tested viruses encoding a Red antigenic motif and 1/7 NYSNYK viruses, but demonstrated >8‐fold loss in cross‐reactivity with the remaining contemporary viruses (Figure 4). Antisera to the Red cluster representative NY/11 were relatively more cross‐reactive with viruses encoding NYSNYK, SYKNYK, and KYNNSK motifs, as well as all but one of contemporary viruses encoding a Green antigenic motif (Figure 4). However, NY/11 antisera had a significant loss in cross‐reactivity with strains encoding the more recently detected Peach (KYHNNK and KHHNNK) or KHNNHK motifs. Antisera to the Green cluster representative IA/14 were cross‐reactive with strains encoding KYNNSK, KHNNHK, and KYHNYK motifs, as well as a single strain encoding a Red antigenic motif, but demonstrated reduced cross‐reactivity to 8/9 strains encoding a Red antigenic motif and those encoding Peach, NYSNYK, and SYKNYK antigenic motifs (Figure 4).

### 3.5 Antigenic phenotype was not restricted to monophyletic clades

**Figure 5:**
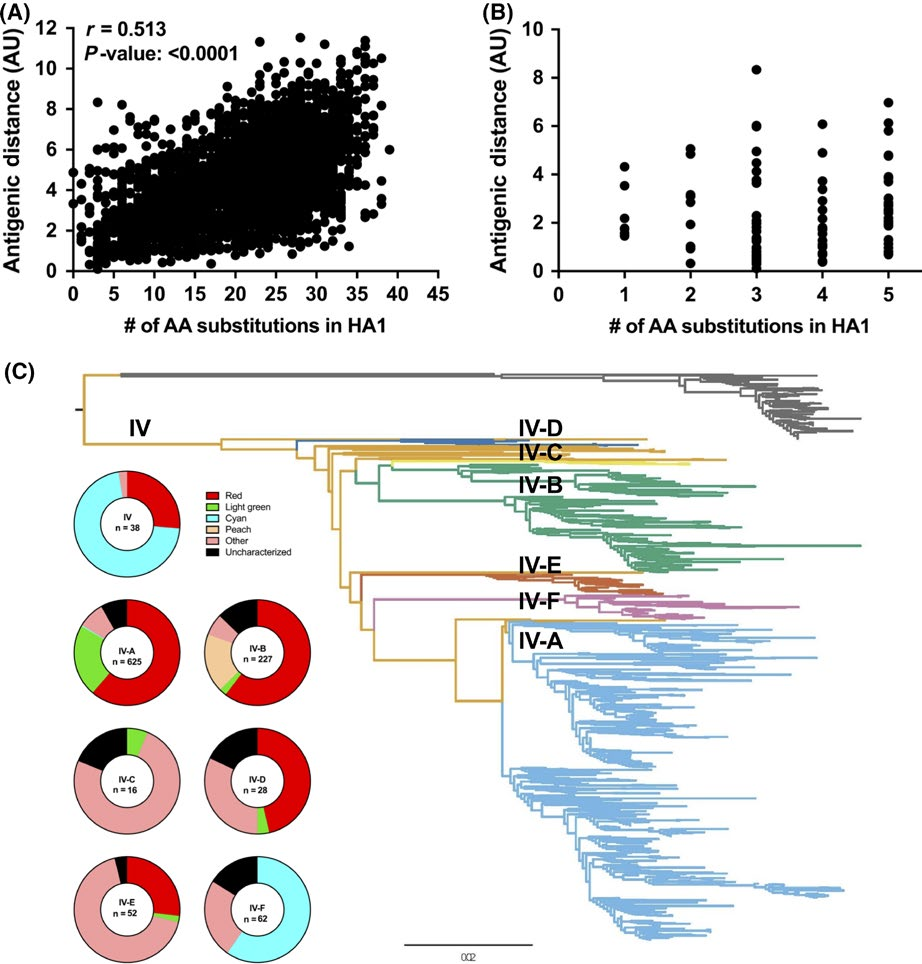
Antigenic and genetic relationships of H3 C‐IV IAV‐S. (A) Pairwise correlation of the antigenic distance between two antigens and the number of amino acid differences in the HA1 between the two antigens. (B) Pairwise correlation from A zoomed in to antigen pairs with five or less amino acid differences in the HA1 region. (C) Distribution of antigenic motifs within C‐IV phylogenetic clades A‐F

The number of substitutions in the HA1 region between each pair of antigens was plotted against the antigenic distance between each pair (Figure 5A). There was a positive linear association between the number of HA1 amino acid differences and antigenic distance (*r* = 0.51, *P* < 0.0001, Pearson's correlation); however, there was a large amount of unexplained variation, supporting the proposition that certain amino acids have a disproportionate impact on antigenic phenotype. We observed marked variability among pairs of antigens that differed by five or less sites in the HA1 region (Figure 5B), often implicating amino acid positions of known importance (Table S3). For example, A/swine/Indiana/A00968373/2012 (NYNNYK) and A/swine/Nebraska/A01478775/2015 (KYNNYK) differed only at position 145 in the HA1 region and were 4.3 AU apart. The inverse of these relationships was also observed, such as in the case of A/swine/Minnesota/A01277201/2012 and A/swine/North Carolina/A01476722/2014 which had 19 amino acid differences in the HA1 but were only 1.7 AU apart and both encoded a KYHNYK antigenic motif.

The distribution of antigenic clusters among the C‐IV clades A‐F was annotated on a maximum‐likelihood tree of H3 genes to assess whether genetic clade^12^ was associated with antigenic phenotype (Figure 5C). Putative antigenic clusters of viruses collected 2012‐2016 were distributed widely across the C‐IV clades, with no single antigenic phenotype populating a single given clade, providing further evidence that a small number of amino acid positions described by the antigenic motif disproportionately affect phenotype, and similarity at these positions is not restricted to monophyletic clades. In addition, these data demonstrate that a high number of antigenically diverse H3 strains are co‐circulating in U.S. swine.

## 4 DISCUSSION

In the 50 C‐IV H3 IAV‐S antigenically characterized here, the number of amino acid combinations within the antigenic motif of wild‐type viruses demonstrated remarkable plasticity. Among the viruses tested with substitutions in the antigenic motif positions, we found that three new motifs (KYHNNK, KHHNNK, and SYKNYK) represented two potentially novel and distinct antigenic clusters. As KYHNNK and KHHNNK demonstrated evidence for sustained circulation during the period of study and into 2017 (n = 11), we designated this motif as the Peach cluster. Although SYKNYK also demonstrated properties of an emerging antigenic cluster, the frequency of detection remained low (no detections in 2017) and assigning an antigenic cluster designation requires evidence of contemporary circulation and additional verification that it is distinct from the Red antigenic cluster. Three of the motifs (NYSNYK, KYNNSK, and KYHNYK) represented additional diversity within previously defined antigenic clusters. One motif (KHNNHK) did not yield a cohesive antigenic phenotype. With a low frequency of detection in recent years (no detections in 2016 or 2017), we did not further explore the diversity in viruses encoding a KHNNHK motif, but further testing of strains and generation of antisera might be warranted if the KHNNHK motif pattern re‐emerges in the swine population. Although some motifs appear to be low in frequency of detection at the current time, they may increase and cause widespread outbreaks if maintained in circulation and if population immunity was focused on dominant H3 antigenic clusters.

Contemporary viruses encoding Red and Green antigenic motifs were phenotypically similar to older strains encoding the same motif, but intra‐cluster variation was observed within these two clusters. Despite such intra‐cluster antigenic variation within the Red and Green clusters between 2012 and 2016, we found that high titer antisera raised to early cluster representatives still cross‐reacted with viruses encoding the same antigenic motif. Some antigenic clusters were maintained over the study time frame, particularly the Red antigenic cluster, predominant since 2009.^13^ The sustained transmission of antigenically distinct viruses over the last seven years may be explained by a lack of long‐lived population immunity in swine herds. In addition to non‐standardized strain composition in vaccines, other factors such as a relatively short generation time for pigs, shared facilities by pigs of different ages and immune status, and long‐distance transport of swine via domestic routes and from Canada may all hinder the acquisition of effective population immunity to strains of a particular antigenic cluster.^21^, ^22^, ^23^

In addition to the sustained circulation of H3N2 viruses in the swine population and introduction of new human H3N2 to swine, there is a continued risk of transmission of H3N2 from swine back to the human population, exemplified by the H3N2 variant (H3N2v) cases reported in recent years.^24^, ^25^ Hundreds of H3N2v cases from 2011 to 2012 resulted from infection with C‐IV viruses from the Red antigenic cluster, and a more recent variant case in late 2016 resulted from infection with a virus from our newly defined Peach antigenic cluster (encoded a KYHNNK motif). However, the majority of the H3N2v cases in 2016 and 2017 were of the 2010 human‐like H3N2 clade in swine.^26^ It remains uncertain whether the C‐IV H3 IAV‐S will be replaced by the more recent human‐like introduction, or if viruses from multiple genetic clades will co‐circulate similar to what is seen with the multiple lineages and clades of H1 IAV‐S.^27^ At the current time, the two H3 lineages continue to co‐circulate. H3 C‐IV viruses remain a useful tool to study influenza antigenic drift due to antigenic heterogeneity, apparent plasticity at the antigenic sites, and availability of isolates through the USDA IAV‐S repository. Ultimately, a better understanding of antigenic evolution of influenza A viruses will help inform vaccine strain selection for more effective vaccines and identify swine strains that potentially pose higher risks when they spill back over to the human population.

## CONFLICT OF INTEREST

None.
