# Supplementary figures and images for: Antigenic evolution of H3N2 influenza A viruses in swine in the United States from 2012 to 2016
Source: Influenza Other Respir Viruses. 2018 Oct 7;13(1):83–90. doi: 10.1111/irv.12610 (PMC6304321; doi:10.1111/irv.12610)

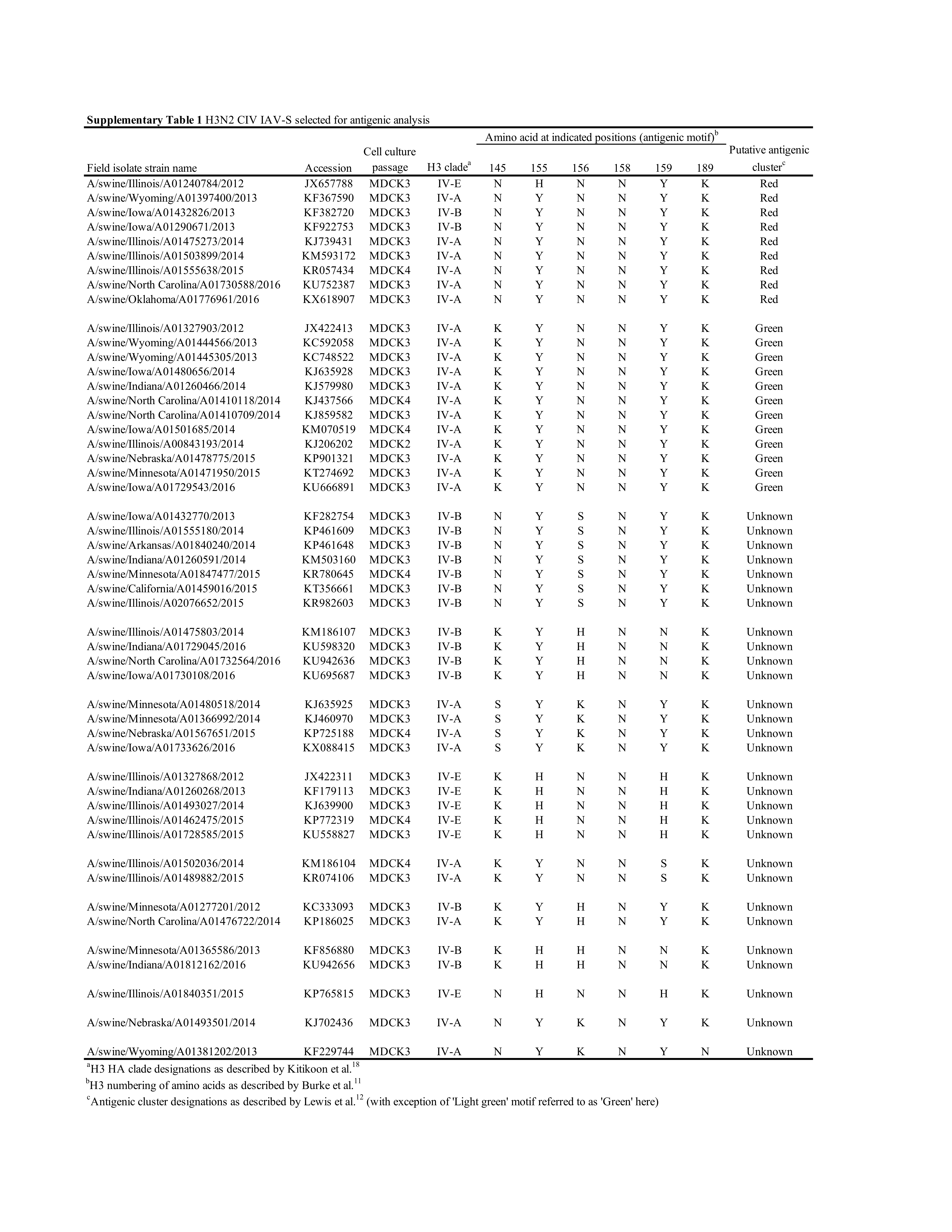

Supplement: Supplementary file 1 [file IRV-13-83-s001.tiff]

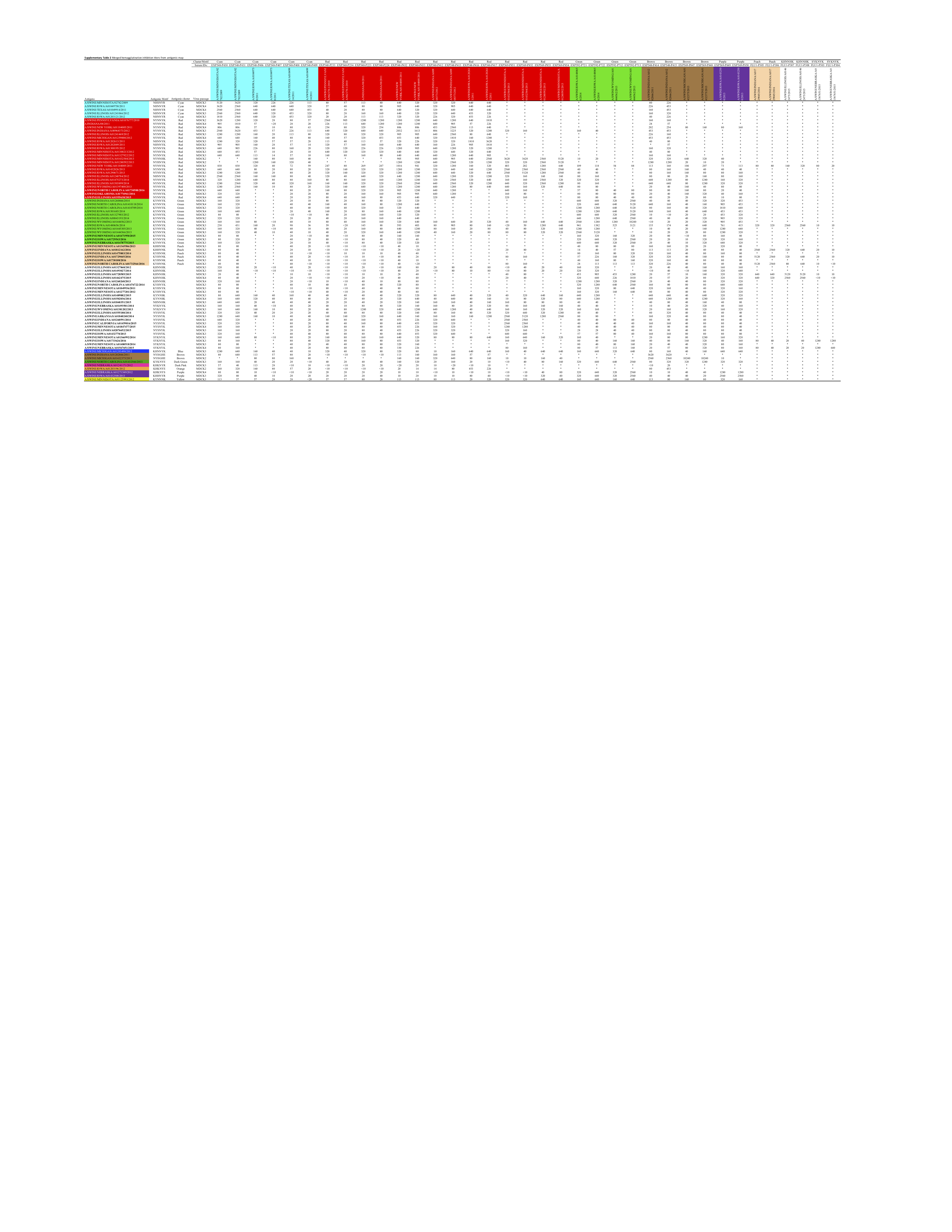

Supplement: Supplementary file 2 [file IRV-13-83-s002.tiff]

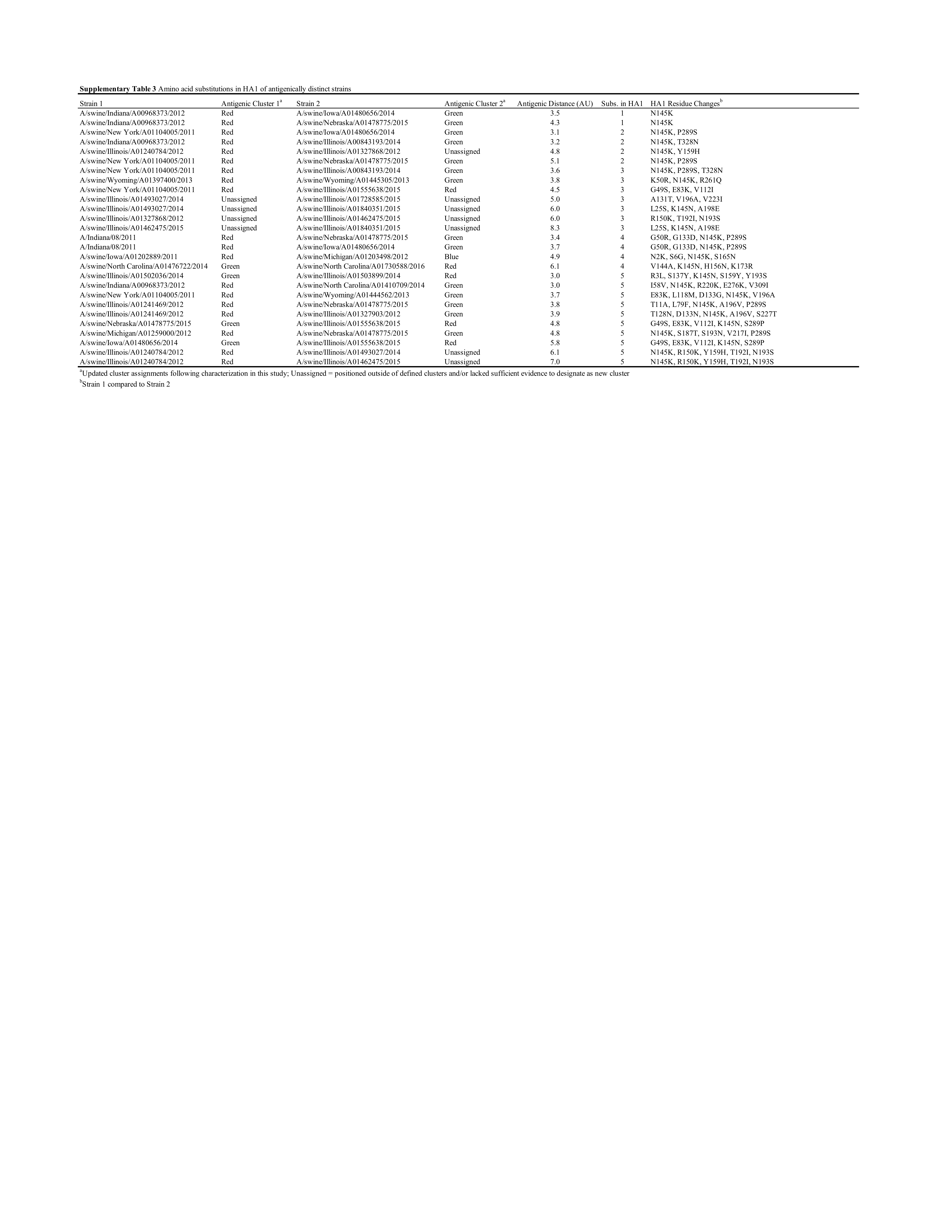

Supplement: Supplementary file 3 [file IRV-13-83-s003.tiff]
